# Why mobile social media-related fear of missing out promotes depressive symptoms? the roles of phubbing and social exclusion

**DOI:** 10.1186/s40359-023-01231-1

**Published:** 2023-06-29

**Authors:** Bin Gao, Quanwei Shen, Gui Luo, Yiwen Xu

**Affiliations:** 1 School of Education, Shanghai Normal University, Shanghai, 200234 China; 2 Mental Health Center, Hefei University of Technology, Hefei, 230009 China; 3 Department of Marxism, Moutai Institute, Renhuai, 564500 China

**Keywords:** Fear of missing out (FoMO), Social exclusion, Phubbing, Depressive symptoms, College students

## Abstract

**Background:**

With the popularity of mobile socialization, people have become more closely connected with their phones. While people enjoy the convenience that phones bring (e.g., accessing information and socializing), they also feel anxious about missing out on certain information. Previous researches have shown that fear of missing out (FoMO) can trigger depressive symptoms, however, the underlying psychological mechanisms are not yet clear. In addition, limited research has explored this issue in the context of mobile social media.

**Methods:**

To address this research gap, we surveyed 486 Chinese college students (278 males and 208 females, mean age = 19.95 years, SD = 1.14) and all participants completed a self-report questionnaire including mobile social media-related FoMO scale, phubbing scale, social exclusion scale, and the patient health questionnaire-9. The data were analyzed by SPSS24.0 and the Process macro and developed a mediating and moderating model incorporating phubbing and social exclusion.

**Results:**

The results showed that (1) mobile social media-related FoMO (MSM-related FoMO) can significantly and positively predict depressive symptoms among college students; (2) phubbing partially mediates the relationship between MSM-related FoMO and depressive symptoms; (3) the direct predictive effect of MSM-related FoMO on depressive symptoms is moderated by social exclusion.

**Conclusion:**

These findings are not only valuable for understanding the underlying mechanisms linking MSM-related FoMO and depressive symptoms, but also contribute to the development of psychological intervention programs (e.g., interventions based on social exclusion or phubbing) aiming at reducing college students’ depressive symptoms.

**Supplementary Information:**

The online version contains supplementary material available at 10.1186/s40359-023-01231-1.

## Introduction

As of June 2022, the number of mobile internet users in China has reached 1.047 billion, and the proportion of mobile Internet users was 99.6% [1]. While smartphones have brought many conveniences to people (e.g., online learning and chatting), their undesirable effects (e.g., phubbing and fear of missing out) have become increasingly prominent and have attracted the attention of researchers [2–5]. The term “fear of missing out” (FoMO) was originally coined by American writer Annie Stamell in a news article in 2011 [6]. Przybylski et al. defined it as a diffuse anxiety caused by individuals’ fear of missing others’ novel experiences or positive events, characterized by the desire to stay continually connected with what others are doing [7]. A review article indicates that FoMO is widely present in both online and offline contexts, 78.3% of Chinese respondents report continuous engagement in various social activities (such as frequent refreshing Micro-blog and attending gatherings) due to FoMO, while 15.2% report severe FoMO symptoms [8]. With the popularity of smartphones, FoMO related to mobile social media (such as Facebook, Instagram, and WeChat) has become increasingly common. We call it mobile social media-related FoMO (MSM-related FoMO), which specifically refers to the anxiety caused by the fear of missing relevant content on mobile social media [9] to distinguish the broadly concept of FoMO [7], online social media-related FoMO and social media-induced FoMO (including smartphones and computers) [10, 11]. Previous studies have found that FoMO is an important source of negative emotions such as depressive symptoms [8, 12], but the underlying mechanisms through which FoMO affects depressive symptoms are still unclear. In addition, limited research has explored this issue in the context of mobile social media. It is worth noting that emerging adulthood university students are not only the main force in mobile social media use, but also face many changes and challenges in life, making them a high-risk group for depression [13]. Therefore, this study aims to explore the mechanism of MSM-related FoMO on depressive symptoms in college students, in order to provide empirical evidence for the prevention and intervention of depression among college students.

### MSM-related FoMO and depressive symptoms

The growing prevalence of smartphones with their increasingly advanced features has led to a rise in people’s attachment to their devices [14]. Concurrently, the occurrence of FoMO in mobile social media has become increasingly prevalent, posing a significant risk for the development of mental health issues [15, 16]. FoMO has been identified as a potential vulnerability factor associated with the onset of depression [12]. Drawing upon the diathesis-stress model of depression [17], it can be argued that individuals with inherent vulnerabilities, such as FoMO, are more susceptible to negative developmental outcomes, including depression. The detrimental effects of FoMO on individuals’ cognition, emotions, and mental health have been well-documented. Firstly, individuals experiencing FoMO may engage in rumination, constantly fixating on what they are missing out on [18], thereby fueling negative thought patterns and exacerbating depressive symptoms. Secondly, FoMO has been linked to various negative emotions, such as stress, anxiety [7], boredom [2], and loneliness [16], which can contribute to depressive symptoms. Furthermore, extensive research has revealed the adverse effects of FoMO on individuals’ mental, physical, and social functioning. These effects encompass disruptions to studying, undermined academic performance [19], reduced self-esteem and life satisfaction [8, 20], compromised sleep quality due to increased cognitive pre-sleep arousal and increased time expended on social media during nighttime [21], and the promotion of problematic internet technology usage [22]. Consequently, we hypothesize that MSM-related FoMO positively predicts depressive symptoms in college students (H1).

### Mediating role of Phubbing

Phubbing refers to the behavior of individuals who are engrossed in using their phones and ignore the people and things around them in social situations [23, 24]. As a common phenomenon in the era of mobile internet, this behavior is widely observed in family, work, and study environments [25–28]. Theory of compensatory internet use suggests that unfulfilled psychological needs in one’s real life can trigger craving for social media on the internet, and negative life situations (e.g., anxiety) can stimulate motivation to go online to relieve negative feelings [29]. However, individuals with FoMO crave to keep up with what others are doing and promptly learn about external information. Therefore, mobile phones, as an important medium that connect others and the world, naturally become their preferred choice. Researches show that individuals with FoMO have higher online vulnerability [30], exhibit more maladaptive social media use (e.g., social media fatigue, problematic smartphone use, and smartphone addiction) [3, 11, 31, 32]. More directly, FoMO can significantly predict individuals’ phubbing [15].

According to David and Roberts [33], phubbing can be considered a form of interpersonal neglect, which has negative consequences for individuals’ social lives. For example, in terms of interpersonal relationships, partner phubbing has been shown to reduce satisfaction in romantic relationships [34]; In terms of happiness, parental phubbing can contribute to adolescents’ alienation [35], while supervisor phubbing can increase subordinates’ psychological distress and diminish employees’ identification with their supervisors [25]; In terms of behavior, parental phubbing can increase adolescents’ learning burnout, interpersonal aggression and SNSs addiction [27, 36, 37]. It is worth noting that previous studies have paid more attention to the influence of phubbing on the phubbees (who receive phubbing), few researchers have focused on the influence of phubbing on the phubbers (who exercise phubbing) [38]. Since interpersonal relationships are mutual, phubbing, being a form of interpersonal neglect, may not only harm the mental health of the phubbees but also affect the mental health of the phubbers. In addition, Karada et al. [24] pointed out that phubbing is the sum of many virtual addictions, representing a multidimensional and complex structure that includes addiction to mobile phones, the internet, SMS, social media, and games. Consequently, phubbers may frequently exhibit addiction to mobile phones, which can negatively impact their normal social functioning and interpersonal connections, potentially leading to depressive symptoms. Moreover, Liu et al. [38] further indicated that phubbing among primary and secondary school teachers from Shanxi province in China increased their risk of job burnout and depression. Based on these findings, we hypothesize that phubbing may mediate the relationship between MSM-related FoMO and depressive symptoms among college students (H2).

### Moderating role of social exclusion

Social exclusion refers to the phenomenon and process in which individuals are ignored or rejected by others or groups, and their need to belong and relationship are hindered [39]. As a form of social setback and trauma, social exclusion widely exists in interpersonal environments such as campus and workplace. The need-threat model of social exclusion points out that establishing and maintaining social connection with others is one of the strongest human needs, and social exclusion can threaten individuals’ sense of belonging and relationship needs [40], thereby bringing about many negative effects, such as inducing anxiety, depression, suicidal ideation and other internalized problems [41–43], as well as reactive aggression, dangerous driving behavior and other externalized problems [44, 45]. The diathesis-stress model of depression further points out that the interaction between individual characteristics and external stressors is the decisive factor in inducing depression [17]. Therefore, the impact of FoMO on depressive symptoms may differ depending on the level of external stressor (e.g., social exclusion). Lower social exclusion means more interpersonal connections and social support for individuals, which may prevent FoMO from developing into depression. Based on this, we hypothesize that the direct predictive effect of MSM-related FoMO on depressive symptoms will be moderated by social exclusion (H3).

### The present study

**Fig. 1:**
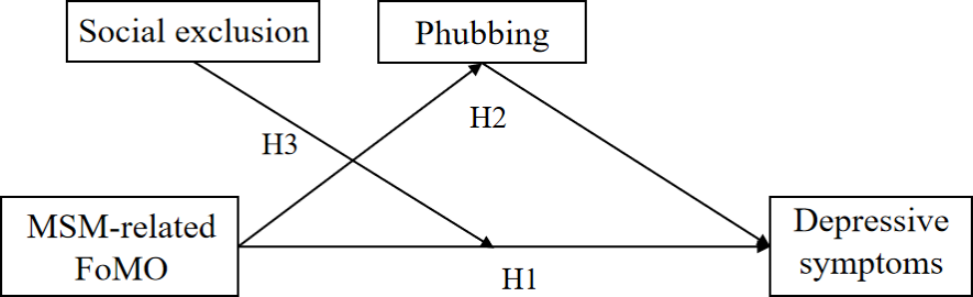
The hypothesized model

In summary, the current study adopted cross-sectional study and constructed a mediating and moderating model (see Fig. 1) to examine: (a) the extent to which MSM-related FoMO would be positively associated with depressive symptoms among Chinese young adults; (b) whether phubbing would mediate the association between MSM-related FoMO and depressive symptoms; and (c) whether social exclusion would moderate the effect of MSM-related FoMO on depressive symptoms.

## Methods

### Participants and procedure

A convenience sampling method was employed to recruit 560 college students from two undergraduate universities in central China to participate in an online questionnaire-based survey. After eliminating invalid responses, a total of 486 valid questionnaires were obtained, resulting in a valid response rate of 86.79%. Of these, 278 (57.20%) were male and 208 (42.80%) were female. The age range of participants was 17 to 25 years old, with a mean age of 19.95 years (SD = 1.14). Among the participants, 41 were freshmen, 357 were sophomores, and 88 were juniors. All participants reported having prior experience with cell phone internet access, with a mean daily online time of 6.49 ± 2.71 h, based on their self-reported estimates.

Data collection occurred during the ongoing COVID-19 pandemic, specifically from April to May 2020. Prior to participating in the online survey, participants provided informed consent. Participation was voluntary, and no financial incentives were provided. The survey took approximately 10 min to complete. This study was approved by the ethics committee at the corresponding author’s institution.

### Measures

#### Mobile social media-related FoMO

The present study utilized the Chinese version of mobile social media-related FoMO scale developed by Song et al. [9] to measure the degree of FoMO. This questionnaire comprises 16 items, divided into four dimensions, namely psychological motivation (e.g., “I feel envious when I see my friends having fun on mobile social media and I am not present”), cognitive motivation (e.g., “Using mobile social media is something I cannot live without in my daily life”), behavioral performance (e.g., “Whenever I have time, I always open mobile social media to see if there are any new news or updates”), and emotional attachment (e.g., “Using mobile social media often makes me feel more fulfilled in my life”). Respondents rated each item on a 5-point Likert scale (1 = “strongly disagree”, 5 = “strongly agree”), with higher scores indicating greater levels of FoMO. The alpha coefficient of this questionnaire in the present study was 0.89.

#### Phubbing

We employed the phubbing scale developed by Karadağ et al. [24] to assess phubbing behavior, which comprises ten items (e.g., “The first thing I do when I wake up in the morning is check my phone”). Participants rated each item on a 5-point Likert scale, ranging from 1 (never) to 5 (always). Prior research has shown that this scale has been validated and can be applied to Chinese college students [46]. Higher scores indicate higher levels of phubbing. The internal consistency coefficient of this questionnaire in our study was 0.88.

### Social exclusion

The study used the Chinese version of the Social Exclusion Scale revised by Zhang et al. (2018) [47] to measure social exclusion. The scale comprised of 11 items that assessed two dimensions of neglect (e.g., “Others often ignore me as if I do not exist”) and rejection (e.g., “Others often invite me to go on vacation with them”). Participants responded to each item on a 5-point scale, where 1 denoted “very non-conforming,“ and 5 denoted “very conforming.“ Higher scores on the scale indicate higher levels of social exclusion. The internal consistency coefficient of the scale in this study was 0.76.

### Depressive symptoms

The Patient Health Questionnaire-9 (PHQ-9) was utilized to assess the subjects’ depressive symptoms [48]. This one-dimensional scale comprises nine items (e.g., “feeling down, depressed, or hopeless”), and respondents rate their experiences on a 4-point Likert scale ranging from 1 (not at all) to 4 (nearly every day). The total score is computed by summing the scores of each item, and higher scores indicate greater levels of depressive symptoms. The PHQ-9 has demonstrated sound reliability and validity among Chinese college students [49]. In the present study, the internal consistency of the scale was excellent, with a Cronbach’s α of 0.90.

### Data analysis

Prior research has indicated that depression may be influenced by gender and daily cell phone use [50, 51]; therefore, we included both factors as control variables in our regression analysis. Similar to the study conducted by Lepp et al. [52], daily cell phone use was assessed using a single question: Please estimate the total amount of time you spend using your mobile phone each day, including activities such as calling, texting, sending photos, gaming, surfing the internet, watching videos, and more. Participants provided best estimates for hours of cell phone use per day. We first calculated descriptive statistics and bivariate correlations among the study variables. To examine the mediation and moderation effects, we utilized the PROCESS macro in SPSS 24.0 [53]. A simple mediation model (Model 4) was employed to assess Hypothesis 2, while Hypothesis 3 was evaluated using Model 5. All continuous variables were standardized before conducting the regression analyses. The significance of the findings was determined using the bias-corrected percentile bootstrap method with 5,000 resamples and a 95% confidence interval (CI). We considered the model statistically significant when the 95% CI excluded zero.

### Common method bias analysis

To address potential common method bias, Harman’s one-factor test was performed on the self-reported data used in this study. Unrotated principal component factor analysis was applied to all items of the study variables. The results showed that there were nine factors with eigenvalues greater than one, and the first factor variance contribution rate was 23.63%, which was lower than the recommended threshold of 40%. Thus, the findings suggest that common method bias is not a serious issue in this study.

## Results

### The measurement model

In our study, we conducted a comprehensive evaluation of the measurement model’s fit using established goodness-of-fit indices, including the chi-square test, comparative fit index (CFI), Tucker-Lewis index (TLI), root mean square error of approximation (RMSEA), and standardized root mean square residual (SRMR). These indices are widely recognized in the field as indicators of model fit. We employed Mplus 8.3 software to evaluate the overall fit of the measurement model. As shown in Table 1, the goodness-of-fit indices for all scales demonstrated acceptable construct validity. Specifically, the CFI and TLI values exceeded the recommended threshold of 0.90, indicating a good fit between the proposed measurement model and the observed data. Additionally, the SRMR and RMSEA values did not exceed the recommended threshold of 0.08, further supporting the adequacy of the measurement model [54]. These findings provide evidence for the satisfactory fit of the measurement model in our study.

**Table 1:** The goodness of fit of the measurement model

	χ2/df	CFI	TLI	SRMR	RMSEA
1.MSM-related FoMO Scale	3.21	0.93	0.91	0.060	0.068
2.Phubbing Scale	3.30	0.96	0.93	0.050	0.075
3.Social exclusion Scale	3.86	0.95	0.93	0.080	0.078
4.The Patient Health Questionnaire-9	3.25	0.97	0.96	0.026	0.069

### Preliminary analysis

As is shown in Table 2, the present study revealed significant positive correlations between MSM-related FoMO and phubbing as well as between MSM-related FoMO and depressive symptoms. Additionally, phubbing was significantly positively correlated with depressive symptoms, and social exclusion was significantly positively correlated with depressive symptoms.

**Table 2:** The descriptive statistics and correlation matrix for each variable

	*M* (*SD*)	2	3	4	5	6
1.Gender						
2.Daily cell phone use	6.49 (2.71)	1				
3.Phubbing	2.49 (0.58)	0.33^**^	1			
4.MSM-related FoMO	3.24 (0.60)	0.24^**^	0.64^**^	1		
5.Social exclusion	2.74 (0.52)	0.08	0.24^**^	0.12^**^	1	
6.Depressive symptoms	1.63 (0.58)	0.17^*^	0.44^**^	0.35^**^	0.26^**^	1
Note: Gender is a dummy variable (0 = male, 1 = female), ^*^p < 0.05, ^**^p < 0.01, ^***^p < 0.001.						

### The mediation and moderation analysis

In order to assess the presence of multicollinearity in our regression analysis, we calculated the variance inflation factor (VIF) for each predictor variable. The VIF values ranged from 1.06 to 1.79, all VIF values are below 5, indicating that multicollinearity was not a serious concern in our analysis [55]. The skewness and kurtosis values for all variables were assessed, and all values fell within the acceptable range of -2 to + 2 for skewness and − 7 to + 7 for kurtosis, indicating a normal distribution of the data [56].

**Fig. 2:**
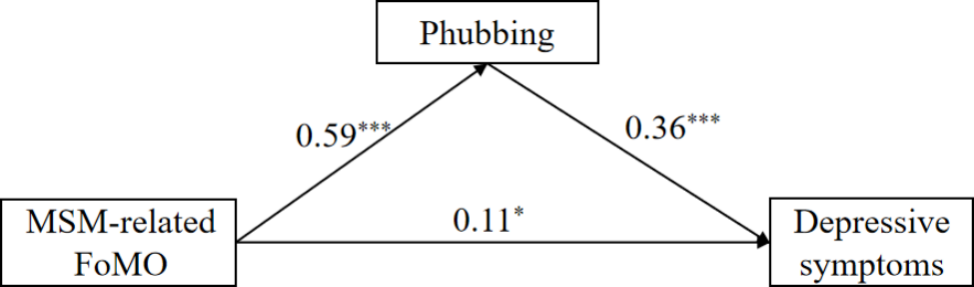
The mediating effect of phubbing. Note.^*^p < 0.05, ^***^p < 0.001

To test Hypothesis 2, we performed a mediation analysis using Model 4 from the Process macro, with gender and daily cell phone use included as covariates. Results (see Fig. 2) showed that MSM-related FoMO not only had a direct effect on depressive symptoms (β = 0.11, *p* < 0.05), but also significantly predicted phubbing (β = 0.59, *p* < 0.001), and phubbing significantly predicted depressive symptoms (β = 0.36, *p* < 0.001). Furthermore, the Bootstrap 95% confidence interval for the mediating effect was [0.14, 0.29], which did not contain 0, indicating that the mediating effect of phubbing was significant. Therefore, Hypothesis 2 was supported. The mediating effect (0.21) accounted for 63.63% of the total effect (0.33).

**Fig. 3:**
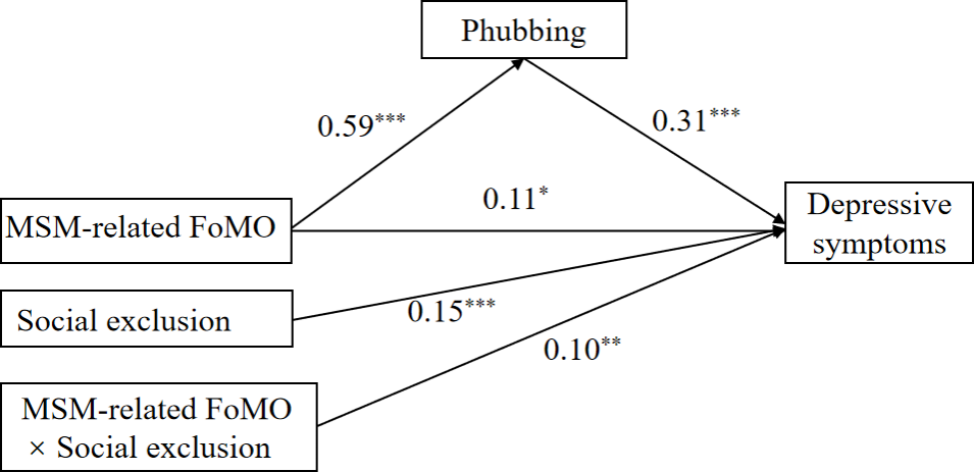
The mediating role of phubbing and moderating role of social exclusion. Note.^*^p < 0.05, ^***^p < 0.01, ^* **^p < 0.001

To test Hypothesis 3, we conducted moderated mediation analysis using Model 5, controlling for gender and duration of cell phone use. Results (see Fig. 3) showed that the interaction between MSM-related FoMO and social exclusion was a significant predictor of depressive symptoms (β = 0.10, *t* = 2.92, *p* < 0.01). The Bootstrap 95% confidence interval for the interaction effect was [0.04, 0.17], which did not contain 0 (see Table 3). This suggests that social exclusion moderated the direct effect of MSM-related FoMO on depressive symptoms, providing support for Hypothesis 3.

**Table 3:** Testing the research hypothesis model

Outcome	Predictors	*R* ^2^	*F*	*β*	*t*	95% CI
Phubbing		0.45	128.40^***^			
	Gender			– 0.04	0.68	[–0.18, 0.10]
	Daily cell phone use			0.07	5.30^***^	[0.04, 0.10]
	MSM-related FoMO			0.59	17.08^***^	[0.53, 0.67]
Depressive symptoms		0.24	24.96^***^			
	Gender			– 0.05	– 0.02	[–0.18, 0.16]
	Daily cell phone use			0.04	3.79	[–0.01, 0.05]
	Phubbing			0.31	5.66^***^	[0.20, 0.42]
	Social exclusion			0.15	3.73^**^	[0.07, 0.24]
	MSM-related FoMO			0.11	2.02^*^	[0.01, 0.21]
	MSM-related FoMO × Social exclusion			0.10	2.92^**^	[0.04, 0.17]

Note: Gender is a dummy variable (0 = male, 1 = female), ^*^p < 0.05, ^**^p < 0.01, ^***^p < 0.001.

**Fig. 4:**
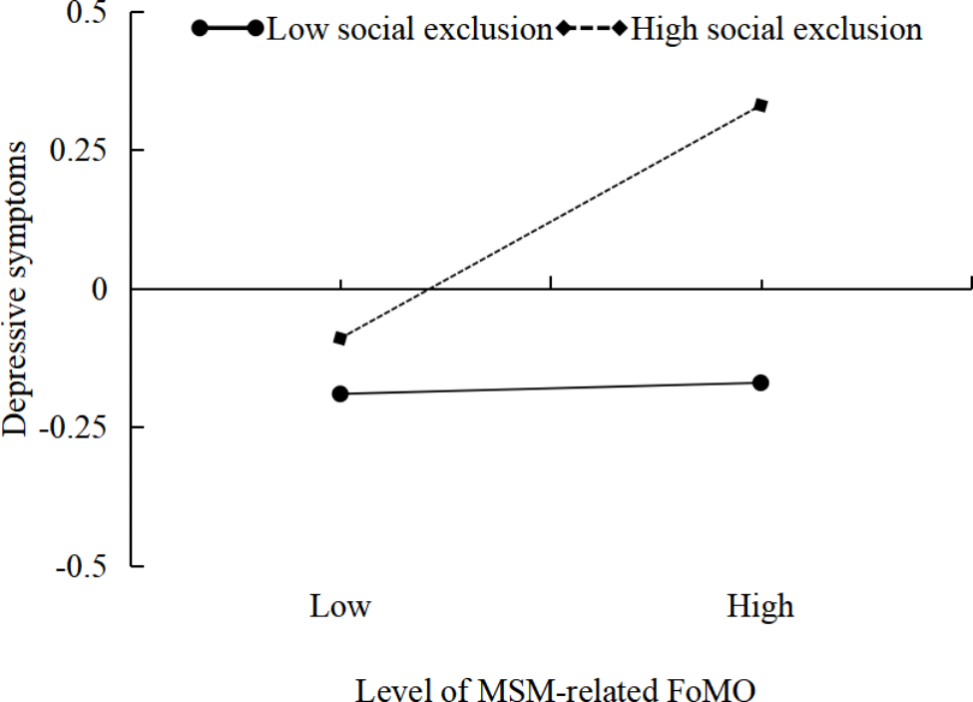
The moderating effect of social exclusion

In this study, we used the pick-a-point method to divide MSM-related FoMO levels into high and low groups based on one standard deviation above and below the mean (M ± 1SD). We then conducted a simple slope analysis to examine the relationship between FoMO and depressive symptoms. Our findings, as shown in Fig. 4, revealed that when social exclusion was low, the change in depressive symptoms levels with increasing FoMO was not significant (β_simple_ = 0.01, *t* = 0.12, *p* > 0.05, 95% CIs: – 0.08 ~ 0.14). However, when social exclusion was high, there was a significant increase in depressive symptoms levels with increasing FoMO (β_simple_ = 0.20, *t* = 3.40, *p* < 0.001, 95% CIs: 0.08 ~ 0.32). These results suggest that the impact of MSM-related FoMO on depressive symptoms is stronger among individuals who experience higher levels of social exclusion.

## Discussion

With the popularity of mobile socialization, FoMO has emerged as a major concern. Prior studies have established a close link between FoMO and depressive symptoms, but the underlying psychological mechanisms are not yet clear. In addition, limited research has explored this issue in the context of mobile social media, namely, MSM-related FoMO. As a diffuse anxiety, MSM-related FoMO is more likely to cause problematic smartphone use such as phubbing, which are undoubtedly risk factors for depressive symptoms. Moreover, individuals’ FoMO and phubbing may have negative effects on their normal social functioning and interpersonal connections, leading to social exclusion. Therefore, this study employs the diathesis-stress model of depression as a theoretical framework to explore the impact of individuals’ variable (i.e., FoMO) and external stressor (i.e., social exclusion) on depressive symptoms. Our results confirmed the positive predictive effect of MSM-related FoMO on depressive symptoms, and further revealed the mediating effect of phubbing and the moderating effect of social exclusion, supporting hypotheses 1–3. The main findings are discussed in greater detail in the subsequent sections.

The results of our correlation analysis indicate a positive association between MSM-related FoMO and depressive symptoms, which is consistent with prior cross-sectional research [12, 57]. There are several possible explanations for this finding. At the cognitive level, FoMO may impair an individual’s positive metacognitive functioning [58], leading to the development of social media addiction. At the affective level, higher FoMO may result in a diffuse anxiety state, which is more likely to induce negative emotions such as anxiety and depression in individuals [50]. At the behavioral level, FoMO may lead individuals to engage in maladaptive behaviors such as problematic cell phone use [12], drinking behavior [59], and poor sleep [60]. Thus, it is clear that FoMO has a negative impact on individuals’ cognition, mood, and daily behaviors, which in turn is likely to exacerbate depressive symptoms.

As mentioned above, few researchers have focused on the influence of phubbing on the phubbers. This study found that there was a positive correlation between the phubbing of phubbers and their own depressive symptoms, which expanded the relevant research. The results show that 20.61% of the variance for college students’ depressive symptoms could be explained by this mediation model. This result supports theory of compensatory internet use [29], which posits that individuals with FoMO are motivated to use mobile social media to alleviate negative emotions and satisfy their psychological needs for external change. Furthermore, FoMO has been described by Przybylski et al. [7] as a phenomenon of self-regulatory failure. Empirical study has also shown that FoMO is linked to self-control failure on social media among college students [61]. Therefore, individuals with high levels of FoMO may lead to ego depletion from constantly browsing mobile social media, impairing self-control and inducing phubbing. As a sum of many virtual addictions, phubbing often involves the weakening of self-control and excessive use of mobile phone [24]. Therefore, phubbers are often addicted to mobile phone, which can damage their normal social functioning and interpersonal connections, thus inducing depressive symptoms. Together, the findings highlight the understanding of underlying mechanisms linking FoMO, phubbing, and depressive symptoms.

The current study identified a moderating role of social exclusion in the relationship between MSM-related FoMO and depressive symptoms, as demonstrated by the fact that the association between MSM-related FoMO and depressive symptoms is stronger among individuals experiencing higher levels of social exclusion. Our results support the diathesis-stress model of depression [17], that is, FoMO is individuals’ vulnerability factor that predisposes individuals to depression, while social exclusion is an external stressor that exacerbates negative emotions. Previous studies have shown that social exclusion has a range of negative effects on individuals’ cognition, emotions, and behavior, making it an important predictor of mental health [62, 63]. Research in brain science has also shown that social exclusion activates the dorsal anterior cingulate cortex, producing a “social pain” that resembles physical pain [64]. From the perspective of the need**-**threat model of social exclusion, social exclusion represents a negative interpersonal experience that threatens individuals’ basic psychological needs, such as belonging, self-esteem, control, and existence [40]. Furthermore, individuals who experience chronic social exclusion may become alienated, helpless, worthless, which increases their risk of negative emotions such as depression [40, 65, 66]. Therefore, lower social exclusion may signify more interpersonal connections and social support, which can alleviate individuals’ depressive symptoms. In other words, as demonstrated in our study, social exclusion can serve as a moderating factor for depressive symptoms.

### Implications

This study holds both theoretical and practical implications. Theoretically, while previous studies have established a strong link between FoMO and depressive symptoms, there is limited investigation specifically in the context of mobile social media, making this study a valuable contribution to the literature. Additionally, our findings support the diathesis-stress model of depression, where social exclusion acts as an external stressor triggering depression, while FoMO, as a diffuse anxiety, may serve as an individual’s vulnerability factor for depression. In terms of practice, depression among emerging adults is a significant public concern, and our findings provide valuable insights for the development of practical interventions aimed at alleviating depressive symptoms among college students (e.g., interventions based on FoMO or phubbing or social exclusion). Specifically, (a) Conduct group counseling with the theme of rational use of social media, and reduce college students’ FoMO by appropriate social media abstinence. Brown and Kuss [67] found that after 7 days of social media abstinence, individuals’ FoMO and smartphone use significantly decreased, while social connectedness and mental wellbeing significantly increased. (b) We should expand offline activities and enrich campus life to increase college students’ outdoor activity time and face-to-face communication opportunities, encouraging them to experience the joys of direct interaction. At the same time, relevant lectures can be organized to raise awareness about the negative consequences of phubbing [25, 27, 34–37], with the intention of reducing such behavior. (c) For college students who have a smaller social circle or exhibit withdrawn personality traits, providing social support can reduce their sense of exclusion and therefore lower the risk of developing depressive symptoms [68, 69].

### Limitations and future research directions

However, this study also has some limitations. Firstly, as a cross-sectional study, it is difficult to establish causal relationships between variables. Future studies could use longitudinal or experimental designs to address this issue. Secondly, this study did not differentiate between various types of social exclusion, and the effects of real-life social exclusion and online social exclusion on depressive symptoms may differ. Thus, future research could explore these distinctions in greater depth. Lastly, this study only examined individual risk factors (FoMO and social exclusion) in relation to depressive symptoms. Future studies could identify protective factors (e.g., self-control, self-compassion, mindfulness) and further examine their impact on depressive symptoms.

## Conclusion

In summary, our study extends current research findings in the following aspects: (a) MSM-related FoMO could positively predict depressive symptoms; (b) phubbing mediated the relationship between MSM-related FoMO and depressive symptoms; and (c) social exclusion can moderate the direct effect of MSM-related FoMO on depressive symptoms. This study enhances our understanding of the mechanisms linking MSM-related FoMO and depressive symptoms, helping college students focus the negative aspect of MSM-related FoMO and use their phones rationally. In addition, our results provide insightful coping strategies for preventing and intervening in depression among college students (e.g., interventions based on social exclusion or phubbing).

## Electronic supplementary material

Below is the link to the electronic supplementary material.

Supplementary Material 1

## Data Availability

The datasets used and/or analyzed during the current study are available from the corresponding author on reasonable request.

## Abbreviations

FoMO: Fear of missing out
MSM-related FoMO: Mobile social media-related FoMO
PHQ-9: Patient Health Questionnaire-9
CI: Confidence interval
CFI: Comparative fit index
TLI: Tucker-Lewis index
RMSEA: Root mean square error of approximation
SRMR: Standardized root mean square residual
VIF: Variance inflation factor
