# Supplementary material for: Why mobile social media-related fear of missing out promotes depressive symptoms? the roles of phubbing and social exclusion
Source: BMC Psychol. 2023 Jun 29;11:189. doi: 10.1186/s40359-023-01231-1 (PMC10311784; doi:10.1186/s40359-023-01231-1)
Supplement: Supplementary file 1 — Supplementary Material 1 [file 40359_2023_1231_MOESM1_ESM.docx]

**Research Scales**

**A. Fear of Missing Out Scale** **in the Mobile Social Media Environment**

1. I feel envious when I see my friends having a good time on mobile social media and I am not there.

2. When I see an alert about a new development on mobile social media, I want to click on it immediately.

3. I always expect someone to chat or "@" me on mobile social media.

4. Using mobile social media is something I can't live without in my daily life.

5. In general, I feel comfortable with the interface design of mobile social media.

6. Using mobile social media makes it easier for me to connect with my family and friends

7. I often use mobile social media to browse articles, music, videos, etc.

8. I use mobile social media to get the information I want about news, business or expertise.

9. Whenever I have time (e.g., waiting for public transportation, taking a break between classes), I often open mobile social media to see if there is any new news or developments.

10. I often use mobile social media to pass the time.

11. I often delay or interrupt my original schedule because of using mobile social media.

12. I need to use mobile social media longer than before to feel satisfied.

13. Using mobile social media often makes me feel that my life is more fulfilling.

14. If I can't use mobile social media for a few days, I feel lost and uncomfortable.

15. Using mobile social media when I am depressed makes me feel better.

16. I have a group of friends with similar interests on mobile social media, like an extended family.

**B. Phubbing Scale**

1. When with others, my eyes pay attention to messages on my phone.

2. When I am with friends, my eyes are always on my phone.

3. My friends complain about my cell phone use.

4. When I am with friends, I am too busy looking at messages on my phone.

5. I don't think I'm bothering my friends when I'm busy with my cell phone messages.

6. My phone is always where I can access it.

7. When I wake up in the morning, the first thing I do is check my phone for messages.

8. I don't feel my day is complete without my phone.

9. I use my phone more and more every day.

10. I have less time for social, personal or professional activities because of my phone.

**C. Social exclusion scale**

1. others will often come to my house to play with me.

2. People often ignore me when we talk together.

3. Others will often invite me to eat with them.

4. Others will always try to get my attention.

5. People often invite me to go on vacation with them.

6. People always ignore my greetings when I meet them on the road.

7. People often invite me to join their clubs, organizations and associations.

8. People often act as if they can't see me.

9. People often invite me to play with them.

10. Others often ignore me as if I don't exist.

11. Others always ignore me.

**D. The Patient Health Questionnaire-9 (PHQ-9)**

1. Can't get excited or uninterested about something.

2. Feeling depressed, frustrated, or hopeless.

3. Difficulty sleeping, insomnia, or excessive sleeping.

4. Feeling tired or lacking energy.

5. Loss of appetite or eating too much.

6. Feeling sluggish or like a failure or letting yourself or your family down.

7. Difficulty concentrating on things like reading the newspaper or watching TV.

8. Moving or talking so slowly that others notice, or the opposite, being irritable or fidgety, moving around more than usual.

9. Thoughts about dying or hurting yourself in some way.
